# Functional near infrared spectroscopy using spatially resolved data to account for tissue scattering: A numerical study and arm‐cuff experiment

**DOI:** 10.1002/jbio.201900064

**Published:** 2019-06-23

**Authors:** Joshua D. Veesa, Hamid Dehghani

**Affiliations:** ^1^ School of Computer Science University of Birmingham Birmingham UK

**Keywords:** near‐infrared spectroscopy, tissue optics, tissue scattering

## Abstract

Functional Near‐Infrared Spectroscopy (fNIRS) aims to recover changes in tissue optical parameters relating to tissue hemodynamics, to infer functional information in biological tissue. A widely‐used application of fNIRS relies on continuous wave (CW) methodology that utilizes multiple distance measurements on human head for study of brain health. The typical method used is spatially resolved spectroscopy (SRS), which is shown to recover tissue oxygenation index (TOI) based on gradient of light intensity measured between two detectors. However, this methodology does not account for tissue scattering which is often assumed. A new parameter recovery algorithm is developed, which directly recovers both the scattering parameter and scaled chromophore concentrations and hence TOI from the measured gradient of light‐attenuation at multiple wavelengths. It is shown through simulations that in comparison to conventional SRS which estimates cerebral TOI values with an error of ±12.3%, the proposed method provides more accurate estimate of TOI exhibiting an error of ±5.7% without any prior assumptions of tissue scatter, and can be easily implemented within CW fNIRS systems. Using an arm‐cuff experiment, the obtained TOI using the proposed method is shown to provide a higher and more realistic value as compared to utilizing any prior assumptions of tissue scatter.
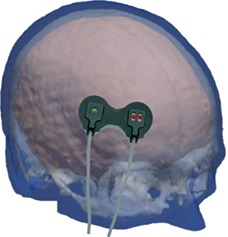

## Graphical abstract

The gradient of attenuation of multi‐spectral Near Infrared light as measured at two or more detectors placed on forehead can provide cerebral tissue oxygenation index by accounting for the subject dependent scattering properties of the underlying tissue.

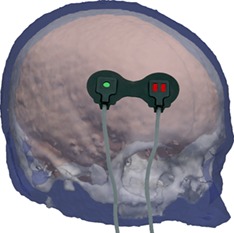

## 1 INTRODUCTION

Prognostic information about brain health has become significantly important in the case of traumatic brain injury (TBI) over the past several years, with TBI being one of the leading causes of death in young people, with up to 20 000 people sustaining a serious brain injury annually 1, 2. The main cause of this high‐mortality rate in TBI (20%–40%) is not due to the initial insult to the brain, but instead it is the variety and complexity of hypoxic, ischemic and cytotoxic events that lead to a profound secondary insult, occurring minutes to hours from the primary event, which poses additional risk to patients 3. Secondary injury can occur at any time after the initial injury including during resuscitation, transport and in the intensive care unit 4. Therefore, an important aspect of improving health‐care for TBI patients is to predict and prevent the secondary injuries, through continuous monitoring of subjects in clinical conditions and taking up the appropriate clinical procedure immediately when required.

Current available brain monitoring modalities for prehospital use are pulse oximetry and blood pressure monitoring systems. The concern with these modalities is that the oxygenation and blood pressure in other tissue is not necessarily an indicative of brain and therefore could lead to incorrect therapy. Clinical methods of brain monitoring include computed tomography, magnetic resonance imaging (MRI), intracranial pressure monitors and electroencephalography 5. The ionizing, invasive or high cost nature of these modalities makes them a weaker tool to be used for the diagnosis of TBI where a continuous monitoring is expected. Near‐infrared (NIR) technology has the potential to fill these gaps and can be used as a nonionizing, noninvasive and a low‐cost alternative to the current methods. NIR light offers a low absorption window in the electro‐magnetic spectrum for biological tissue allowing it to penetrate through the skull and reach the brain. Biological tissues have two dominant optical properties in the NIR window, spectrally varying absorption and scattering. Absorption is caused predominantly by oxy and deoxy‐hemoglobin and water. Scattering is mainly due to collagen, melanin, lipids and keratin present in skin, blood vessels and various soft‐tissues.

In the context of brain monitoring, by transmitting NIR light and measuring the back‐scattered light‐intensity on the scalp it is possible to get spectrally resolved measures of absorption and scattering information about the deeper regions where light has propagated. Using multiple wavelength information (spectroscopy) it is possible to quantify the amount of oxy/deoxy‐hemoglobin present in the tissue and hence its oxygen saturation, that is, the tissue oxygenation index (TOI), being a promising biomarker in monitoring brain health 6. There are two different approaches used to estimate these tissue properties: spectroscopy and imaging. Spectroscopy involves very few source‐detectors for measurements and provides a bulk (whole tissue) estimate of optical properties usually using an analytical estimate of homogeneous semi‐infinite slab. Due to the assumed homogeneity, the estimated optical properties usually suffer from the contamination from superficial layers 7.

The use of multiple overlapping measurements has demonstrated the ability of NIR spectroscopy (NIRS) to image the brain region using tomographic reconstruction techniques 8. These methods can use the anatomical information of different regions of the head derived from other imaging modality such as an MRI, and predict the photon propagation using numerical modeling of the diffusion approximation to model the light intensities for every source‐detector combination and iteratively fit the optical properties of the medium such that the modeled data matches with the measured data 9. As these methods take into consideration the head geometry, tomographic imaging methods tend to give more accurate measure of cerebral optical properties as compared to homogeneous infinite medium approximations. But this would require computations involving hundreds to thousands of measurements depending on the number of sources and detectors, and is generally slow in comparison with spectroscopic techniques, which makes spectroscopy methods more viable for continuous real‐time monitoring of brain health 10.

Based on the type of light‐source and detection system used, NIRS technology is classified into continuous wave (CW), frequency domain (FD) and time‐resolved (TR) systems. While a CW system measures only the attenuated light intensity, FD and TR systems can measure the pathlength of the light traveled through the tissue which allow for a more accurate quantitative assessment of its optical properties. FD systems measure the phase change of light which is an indication of the extent of scattering the light has undergone. Therefore, the phase measurement in addition to the intensity attenuation helps distinguish between absorption and scatter quantitatively. On the other hand, TR systems measure the times of arrival of photons (proportional to pathlength) and their intensities. A TR system is considered to have the greatest accuracy in the quantitative distinction between absorption and scatter and is considered the “gold standard” technique to measure optical properties of tissue 11. However, these additional measurements of pathlength in both FD and TR systems require stable measurements, and have a low signal‐to‐noise ratio in comparison to the CW systems. With CW‐NIRS being more robust and cost‐effective as compared to FD and TR systems 11, it is widely used clinically to monitor cerebral oxygenation 12, 13, 14.

The major challenges with a CW‐NIRS system are to remove any superficial layer contamination from brain signal and to separate absorption and scattering properties. While a single source‐detector measurement samples superficial layer as well as the brain region, an additional short separation measurement can be used to sample just the superficial layer, which can then be regressed from the measured signal to minimize superficial layer contamination 15, 16. Spatially resolved spectroscopy (SRS) is a widely‐used technique in CW‐NIRS developed to obtain TOI from deep tissues, with minimal contamination from superficial layers by measuring the gradient of light‐intensity attenuation at multiple wavelengths using a set of closely placed detectors at nearly the same source‐detector distance, large enough to probe the cerebral tissue 17. This estimation of TOI generally relies on the assumption of scattering parameters of biological tissue which is based on empirical findings 18. Since the underlying scattering properties vary across different subjects and tissue types, this assumption of scattering parameters would lead to uncertainty in the estimated TOI.

In this work, we propose a method that further develops the multi‐distance SRS technique to reduce superficial layer contamination in the estimation of TOI, but at the same time also fit for the underlying unknown scattering properties to remove uncertainty due to inter‐subject variability in scattering properties to derive TOI directly.

## 2 METHODOLOGY

The solution to the CW diffusion equation for a homogeneous semi‐infinite medium is given by, 19

(1) $$I=\frac{I_{0}}{2{\pi \mu }_{s}^{'}\rho^{2}}\left[\sqrt{3\mu_{a}\;\mu_{s}^{\prime }}+\frac{1}{\rho }\right]\exp\left(-\rho \sqrt{3\mu_{a}\;\mu_{s}^{\prime }}\right).$$

This equation describes the intensity of (back‐scattered) light (I) for a source intensity *I*_0_, measured at a distance $\rho >>1/\mu_{s}^{\prime }$ from source, in terms of optical properties of the medium. Here, *μ*_*a*_ and $\mu_{s}^{\prime }$ are the bulk absorption and reduced scattering coefficients of the medium. For the case of a homogeneous medium, this equation is widely used to derive the absorption of the medium from multi‐distance intensity (or attenuation) measurements 20, 21. However, in the case of a complex multi‐layered medium such as the human head, this relation becomes inappropriate, as the recovery of cerebral tissue parameters will always be contaminated by the superficial layers of head, such as skin, scalp, bone and cerebrospinal fluid.

A different formulation is derived from the same equation, which defines the gradient of light‐attenuation *A* = ln(*I*_0_/*I*) at a large source‐detector separation ($\rho >>1/\sqrt{3\mu_{a}\mu_{s}^{\prime }}$) as 17,

(2) $$\frac{\Delta A}{\Delta \rho }=\frac{\Delta \ln\left(I_{0}/I\right)}{\Delta \rho }=\frac{-\Delta \ln\left(I\right)}{\Delta \rho }\approx \sqrt{3\mu_{a}\;\mu_{s}^{\prime }}+\frac{2}{\rho }.$$

**Figure 1:**
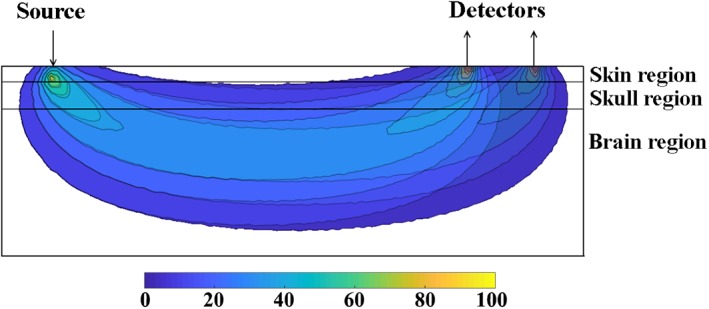
A qualitative schematic showing the sensitivity of light‐attenuation in a three‐layered medium. The two detectors are modeled at 37 and 43 mm from the source

The attenuation gradient *A* is typically measured by two detectors that are closely spaced as compared to their distance from source (eg, detectors separated by 6 mm compared to source‐detector distances of approximately 40 mm for a commercially available NIRO‐200NX system). NIR light is attenuated by the superficial layers as well as the cerebral tissue before reaching the detectors. For large source‐detector separations, the sensitivity of light attenuation due to the superficial layer is similar at these two detectors as demonstrated in Figure 1 and therefore the sensitivity of gradient measurement at these superficial layers becomes much lower.

**Figure 2:**
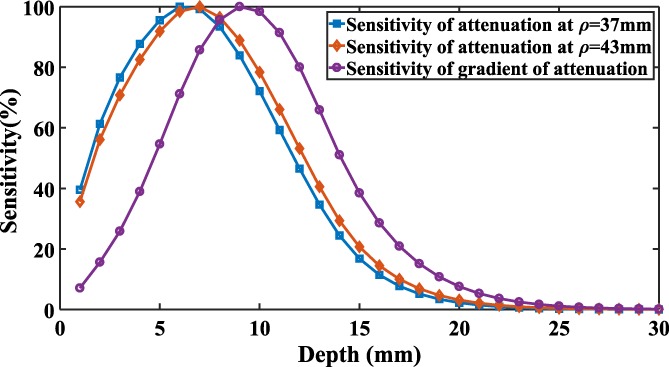
Sensitivity of attenuation with respect to depth for individual detectors as well as the gradient between them

This reduction of light sensitivity to superficial tissue for the gradient of attenuation measurement is better illustrated in Figure 2. Here, the total sensitivity of intensity due to absorption is plotted as a function of depth for both detectors at distances of 37 and 43 mm from the source as well as the gradient of attenuation between them, as simulated using near‐infrared flourescence and spectral tomography (NIRFAST) 8, which is an open source numerical package for modeling diffuse light propagation in biological tissue. This is calculated for a source detector separations of *ρ* = 37 and *ρ* = 43 mm using a three‐layered rectangular mesh (200 × 100 × 100 mm^3^) with mean element size of 0.7 mm^3^, layer‐1 thickness of 5 mm (depicting skin), and layer‐2 thickness of 8 mm (depicting skull), using an average estimate of optical properties of biological tissue, Table 1 22. As evident in Figure 2, the magnitude of the gradient of attenuation is less sensitive to the superficial layer as compared to individual attenuation measurements, which implies that the measured gradient of attenuation is comparatively affected less by the hemodynamics of superficial layers, while showing higher sensitivity to deeper regions.

**Table 1:** Baseline tissue optical parameters 22, 23

Parameter↓	Region→	Skin	Bone	Brain
Total hemoglobin (mM)		0.07	0.049	0.076
Tissue oxygenation index (%)		80	80	50 to 80
Scatter power		1.42	0.72	1.61
Scatter amplitude (mm^−1^)		1.72	1.40	0.80

The main drawback of using the intensity gradient based method is that the absorption coefficient and reduced scattering coefficient are always coupled and cannot be separated. To separate *μ*_*a*_ and $\mu_{s}^{\prime }$ from the gradient measurement, Farrell et al 20 used the additional information of reflectance measured at smaller source‐detector separation to obtain $\mu_{s}^{\prime }$, whereas Liu et al 24 exploited the intercept of log(*ρ*^2^*I*) versus *ρ* to find $\mu_{s}^{\prime }$ and Matcher et al 18 used TR methods to find the scattering coefficient initially and assumed that the scattering properties are similar across same tissues types for all subject. These methods use an additional measure to decouple *μ*_*a*_ and $\mu_{s}^{\prime }$ which would be applicable only for a homogeneous medium. But for a layered medium such as the human head, all these additional measures contain information either from the superficial layer alone or superficial and cerebral tissue combined, and would therefore limit the inherent advantage of probing the deeper layers and being less sensitive to superficial layers, as exhibited by the gradient measurement alone.

A new approach that uses intensity gradient measurements at multiple wavelengths to decouple the absorption and scattering parameters to obtain TOI is presented. Rewriting Equation (2) in terms of scattering parameters and chromophore concentrations (*C*_1_, *C*_2_, *C*_3_ … corresponding to the molar concentrations of the chromophores oxy‐hemoglobin, deoxy‐hemoglobin, water volume fraction and other major light‐absorbing tissue constituents),

(3) $$\frac{1}{3}{\left[\frac{\Delta A\left(\lambda \right)}{\Delta \rho }-\frac{2}{\rho }\right]}^{2}=\mu_{a}\left(\lambda \right)\;\mu_{s}^{\prime }\left(\lambda \right)=\left[\sum_{j=1}^{N}\varepsilon_{j,\lambda }C_{j}\right]\;a\;R\left(b,\lambda \right).$$

The absorption coefficient at a given wavelength is related to the chromophore concentrations (*C*_*j*_) as: $\mu_{a}\left(\lambda \right)=\sum_{j=1}^{N}\varepsilon_{j,\lambda }C_{j}$; where “*ε*_*j*, *λ*_” is the molar extinction coefficient of chromophore‐*j* at *λ* (μm) wavelength and “*N*” is the number of chromophores contributing to absorption of light at the measured wavelengths.

The dependency of the reduced scattering coefficient on wavelength can also be represented as, $\mu_{s}^{\prime }\left(\lambda \right)=a\;R\left(b,\lambda \right)$. Considering all the scattering particles to be spherical, elastic and of similar size 25, the Mie theory approximation fits well for the exponential relation as given by 26,

(4) $$R\left(b,\lambda \right)={\left(\lambda \right)}^{-b}.$$

Due to the practical inhomogeneity in the size, density and type of scattering particles in a tissue, the exponential relation may not be able to accurately represent the scattering spectrum of a tissue. Alternatively, a linear approximation of the variation of $\mu_{s}^{'}$ with respect to wavelength can be used to yield an alternative representation of scattering spectrum 17, 18, which can be expressed as:

(5) $$R\left(b,\lambda \right)=1-\mathrm{b\lambda}.$$

Similar to Equation (2) the chromophore concentrations (*C*_*j*_) and the scattering parameter “*a*” are coupled and would lead to infinitely many solutions. Therefore, with the normalized chromophore concentrations defined as $C_{j}^{\prime }=a\;C_{j}$, Equation (3) can be re‐written as:

(6) $$\frac{1}{3}{\left[\frac{\Delta A\left(\lambda \right)}{\Delta \rho }-\frac{2}{\rho }\right]}^{2}=\left[\sum_{j=1}^{N}\varepsilon_{j,\lambda }C_{j}^{\prime }\right]\;R\left(b,\lambda \right).$$

Considering the left‐hand‐side term of Equation (6) to be γ(λ), the known measurements are γ(*λ*_1_), γ(*λ*_2_) … at multiple wavelengths and the unknown parameter set (**p**) to be recovered is: {*b*, $C_{1}^{\prime }$ *,* $C_{2}^{\prime }$ *,…* $C_{N}^{\prime }$}. Ideally at least *N* + 1 wavelengths are required to solve for these *N* + 1 unknowns (*N* normalized chromophore concentrations and 1 scattering parameter). The inverse problem of solving this nonlinear equation is implemented by an iterative regularized least‐square minimization with the following update equation for each iteration 27,

(7) $$\Delta p={\left(J^{T}J+\alpha \;I\right)}^{-1}J^{T}\;\Delta \gamma .$$

Here, Δ**p** represents the update for the parameter set {*b*, $C_{1}^{\prime }$, $C_{2}^{\prime }$ *,…* $C_{N}^{\prime }$}, Δ**γ** is the data‐model misfit at the end of each iteration and **J** is the Jacobian (or sensitivity) matrix with its structure is given by,

(8) $$J=\left[\begin{array}{cccc} \frac{\partial \gamma \left(\lambda_{1}\right)}{\partial C_{1}^{\prime }} & \frac{\partial \gamma \left(\lambda_{1}\right)}{\partial C_{2}^{\prime }} & \cdots & \frac{\partial \gamma \left(\lambda_{1}\right)}{\partial b}\\ \frac{\partial \gamma \left(\lambda_{2}\right)}{\partial C_{1}^{\prime }} & \frac{\partial \gamma \left(\lambda_{2}\right)}{\partial C_{2}^{\prime }} & \cdots & \frac{\partial \gamma \left(\lambda_{2}\right)}{\partial b}\\ \vdots & \vdots & & \vdots \end{array}\right].$$

The stability of the inverse problem relies on the condition number of the matrix **J** which varies with the values of the parameters given by **p**. The condition number directly relates to the amount of information available for parameter recovery: The higher the condition number corresponds to larger instability and therefore a lower condition number is desired. To further aid the ill‐conditioned problem, the inverse problem is guided with a regularization parameter given by *α*. The Jacobian matrix can be computed using the perturbation method on Equation (6) and as this is an analytical expression, the whole process of the inverse problem can be accomplished in real‐time.

Once the parameter set **p** is recovered, the TOI can then be calculated from the normalized oxy hemoglobin concentration ($C_{1}^{\prime }$) and normalized deoxy hemoglobin concentration ($C_{2}^{\prime }$) as, $\mathrm{TOI}\left(\%\right)=100\times C_{1}^{\prime }/\left(C_{1}^{\prime }+C_{2}^{\prime }\right)$. This algorithm is defined as Spectrally Constrained Spatially Resolved Spectroscopy (SCSRS).

## 3 OPTIMIZATION OF WAVELENGTHS

The stability of the inverse problem relies on the condition number of the sensitivity matrix (**J**). Lower condition number implies a good recoverability of parameters, with low cross‐talk. Remembering that $\mu_{a}\left(\lambda \right)=\sum_{j=1}^{N}\varepsilon_{j,\lambda }C_{j}$, the distinguishability of oxy‐hemoglobin and deoxy‐hemoglobin should also be taken into account and it depends on the extinction coefficient matrix (**E**) given by 28,

(9) $$E=\left[\begin{array}{cc} \varepsilon_{1,\lambda_{1}} & \varepsilon_{2,\lambda_{1}}\\ \varepsilon_{1,\lambda_{2}} & \varepsilon_{2,\lambda_{2}}\\ \vdots & \vdots \end{array}\right].$$

Here, *ε*_**1**_ and *ε*_**2**_ represent the extinction coefficients of oxy‐hemoglobin and deoxy hemoglobin respectively. In this work, the recovery of just two chromophores oxy and deoxy‐hemoglobin is considered, that is, *N* = 2 and therefore the three optimal wavelengths to implement this algorithm are sought. To this end, different combinations of a set of three wavelengths are considered from the spectra 650 to 850 nm with 4 nm separation. The wavelength range is limited from 650 to 850 nm to avoid the peak absorption wavelengths for water and lipids, so that oxy and deoxy hemoglobin remain as the major chromophores contributing to light absorption in this wavelength region.

**Figure 3:**
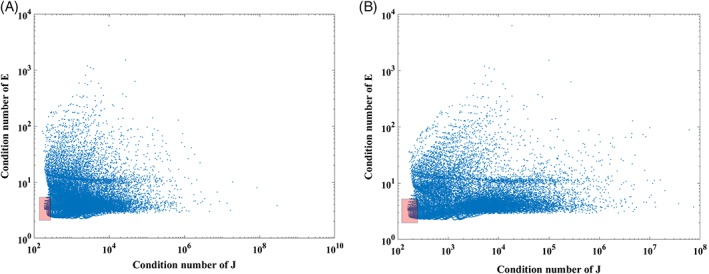
Scatter plot of condition numbers calculated for E and J with (A) exponential and (B) linear model for scatter

The condition numbers of **E** and **J** are calculated for different combinations of wavelengths as shown in Figure 3. The highlighted region shows the combinations with low condition number of **E** (<5.3) and low condition number of **J** (<250).

**Figure 4:**
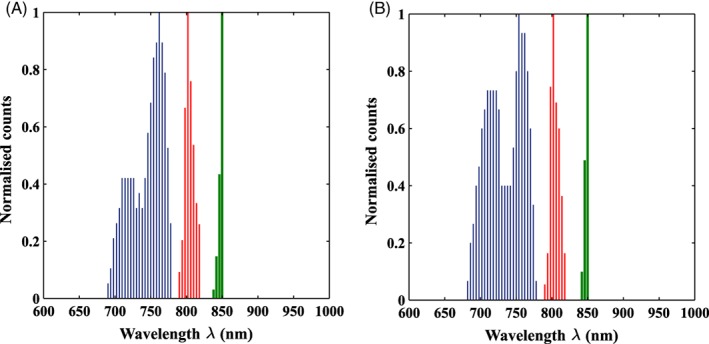
Normalized histogram of optimal wavelengths that satisfy the low condition number criterion for (A) exponential and (B) linear model of scatter

The histograms of optimized wavelength sets that satisfy this low condition number criterion are shown in Figure 4. The optimal three wavelengths for the exponential scattering model are 744 ± 23, 805 ± 7 and 848 ± 2 nm. Similarly, the optimal wavelengths for the linear scattering model are found to be 734 ± 25, 805 ± 6 and 848 ± 2 nm, demonstrating that the optimal wavelengths for both models seem to be similar. This work is concerned with recovering only oxy and deoxy hemoglobin and given the large difference in their respective extinction coefficients at lower wavelengths gives rise to the wide distribution of optimal choice at these lower wavelengths. It is however important to note that the associated condition number of J for any set of three wavelengths within this distribution has minimal variation.

## 4 NUMERICAL VALIDATION ON HEAD MODEL

**Figure 5:**
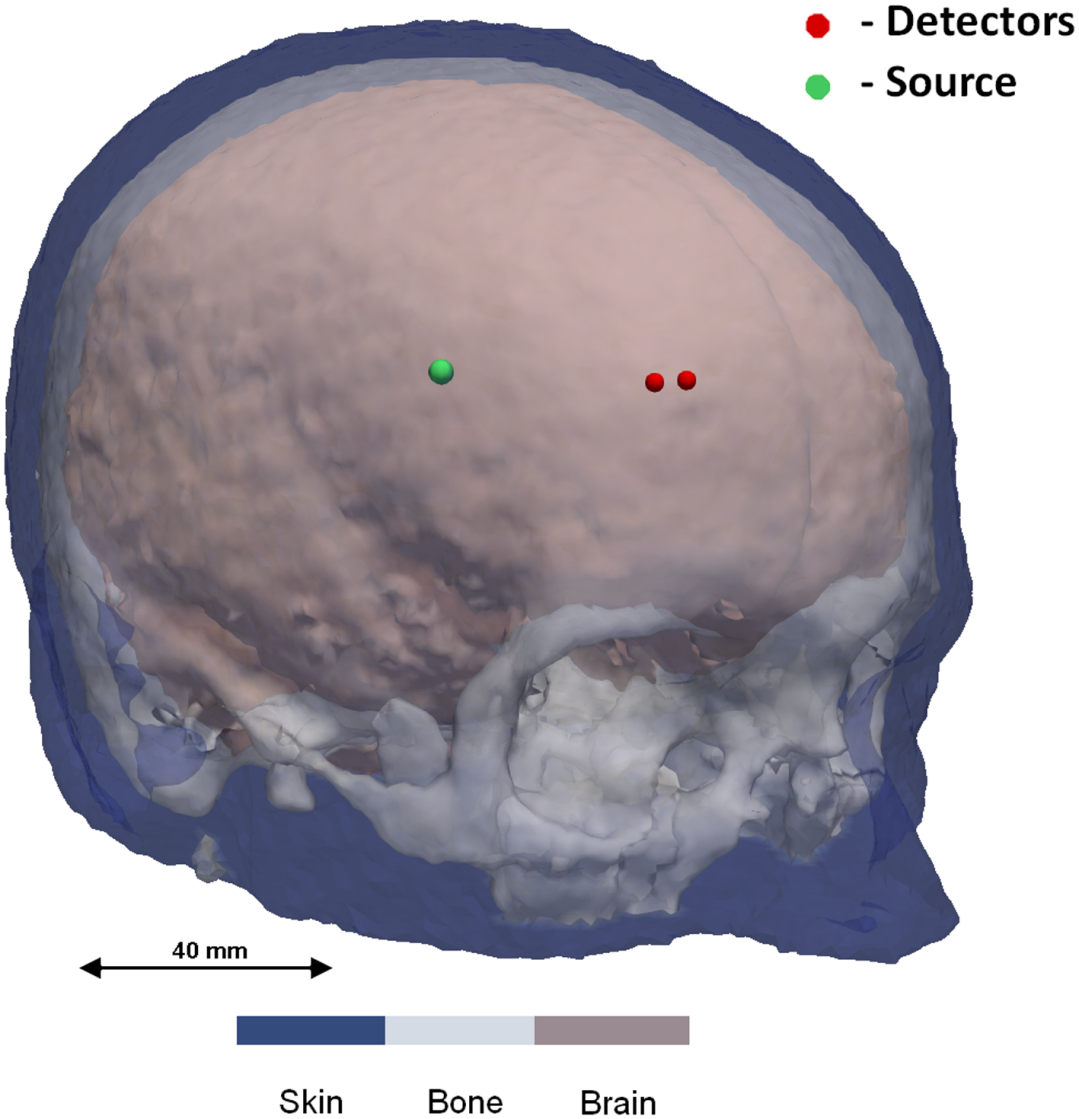
Probe locations shown on a 3‐layered model of human head

MRIs of 10 different subjects are used to build different head models. One source and two detectors (37 and 43 mm from the source) are placed on the forehead as shown in Figure 5, operating at wavelengths 735, 810, 850 nm; this probe model is similar to the commercially available NIRO‐200NX and these wavelengths combination fall within the optimal wavelength set derived in the previous section. Using the baseline properties given in Table 1, the transmitted light intensity data is simulated on NIRFAST. The scatter amplitude and scatter power for three regions shown in Table 1 are randomly distributed around the baseline value by +/−30% to account for inter‐subject variability. Two cases are examined here for data simulation: (a) the medium is assumed to be homogeneous with optical properties of brain to account for zero superficial layer contamination, (b) the medium is considered as a heterogeneous three layered model as shown in Figure 5 to study a more realistic case. The 10 head models used have an average skin thickness of 5 mm and average skull thickness of 8 mm.

**Figure 6:**
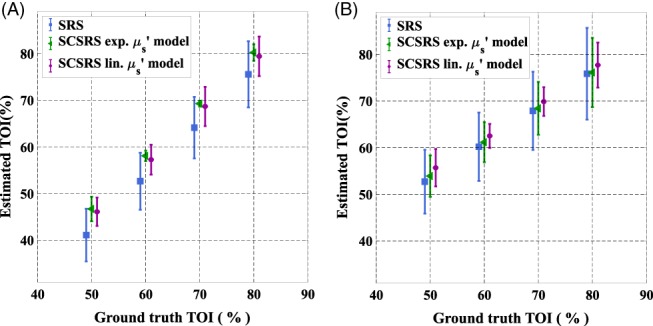
Recovered tissue oxygenation index values of the brain with standard‐deviation of recovery across 10 head models, (A) homogeneous head model, (B) three‐layered head model, using both exponential (exp.) and linear (lin.) scattering models

**Figure 7:**
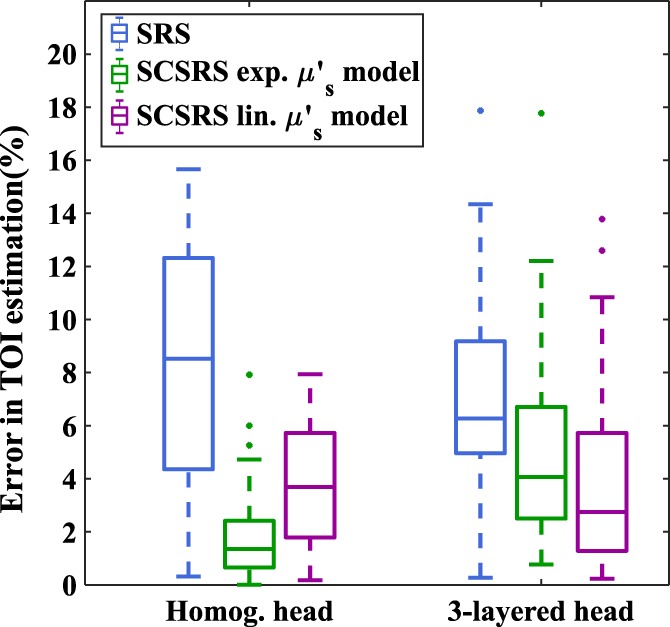
Box‐plot of absolute error in the estimation of tissue oxygenation index (TOI), showing the distribution of errors across different ground‐truth TOI and scattering parameters for, homogeneous and three‐layered head model

The regularization parameter *α* for the inverse problem as shown in Equation (7) is considered as 0.01 times the maximum diagonal value of **J** ^T^ **J**. The recovered TOI values are shown in Figure 6 for the proposed SCSRS (for both exponential and linear scatter model) method as well as those with the regular spatially resolved spectroscopy (SRS), as defined elsewhere 17 with the error in the estimation of cerebral TOI values shown in Figure 7. For the conventional SRS method, Equation (6) is used in combination with the linear scatter model described in Equation (5), along with *b* = 0.63 μm^−1^ as is typically chosen to represent brain tissue 17. With the assumption of *b* parameter, Equation (6) becomes a simple linear equation in {$C_{1}^{\prime }$ *,* $C_{2}^{\prime }$} and can be solved to recover TOI.

**Figure 8:**
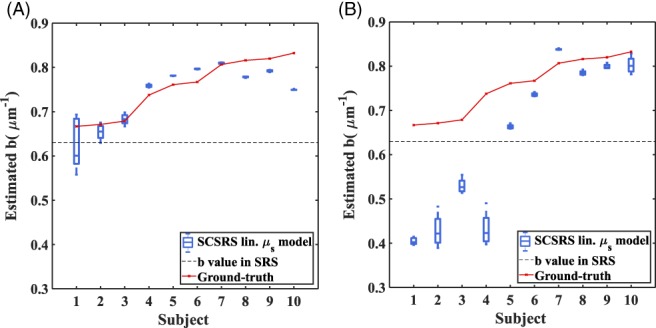
Recovered *b*‐value (corresponding to linear scattering model) with Spectrally Constrained Spatially Resolved Spectroscopy for (A) homogeneous head models, and (B) 3‐layered head models, of 10 different subjects with different scattering parameters. The boxplot shows the distribution of recovered “*b*” for different cerebral tissue oxygenation index (50% to 80%). The solid line represents the ground‐truth values of the homogenous medium in figure (A) and of the cerebral region in figure (B). The dotted line marks the value assumed in spatially resolved spectroscopy method

Figures 6 and 7 show that for both homogeneous and layered head scenarios, SCSRS method has higher accuracy in recovering the cerebral TOI with a maximum third quartile (75th percentile) error of ±5.7% for the linear scatter model (±6.7% for exponential scatter model), compared to that of ±12.3% for SRS method. The lower errors of SCSRS with exponential scatter model for homogeneous head scenario are due to the inverse crime of using the exponential scatter model to simulate the data and recover the parameters as well. However, for the 3‐layered head scenario this advantage is absent due to the inhomogeneity in the scattering properties of the medium, and the homogeneous approximation (since SCSRS in based on a homogeneous model) of a linear scatter model performed better than the exponential scatter model. The recovered values of *b* parameter in SCSRS method corresponding to the linear scatter model is shown in Figure 8 for both homogeneous and 3‐layered head scenarios, for all varying modeled TOI values, in comparison to the ground‐truth values and also the constant approximated *b* value of 0.63 μm^−1^ in SRS method. It should be noted that the ground‐truth scatter model used in simulations is the exponential model, and the values shown in Figure 8 correspond to a linear approximation to the exponential model. This clearly demonstrates the variation in the best‐fit *b* parameter across different subject models of different scattering properties.

## 5 EFFECT OF SUPERFICIAL LAYER THICKNESS

Although the recovered brain TOI values using this proposed gradient based method are shown to be less sensitive to variations in scattering, their accuracy in the recovery of cerebral tissue parameters is limited due to the heterogeneous layered nature of the head. Here the effect of superficial layer thickness in determining cerebral TOI is demonstrated using the proposed SCSRS method and compared to the conventional SRS.

**Figure 9:**
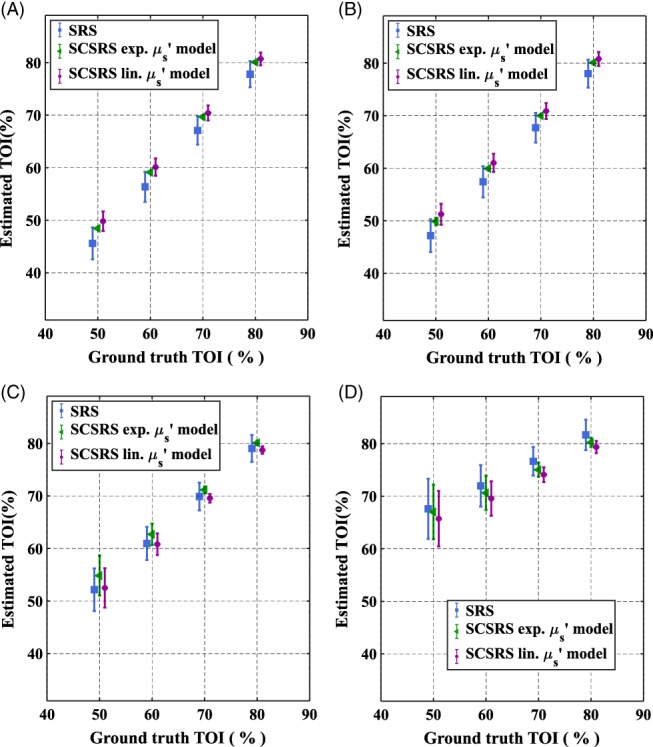
Recovered tissue oxygenation index values with standard‐deviation of recovery across 30 different cases of randomly varying scattering properties, for a skin + skull thickness of (A) 0 mm, (B) 4 mm, (C) 8 mm and (D) 14 mm

**Figure 10:**
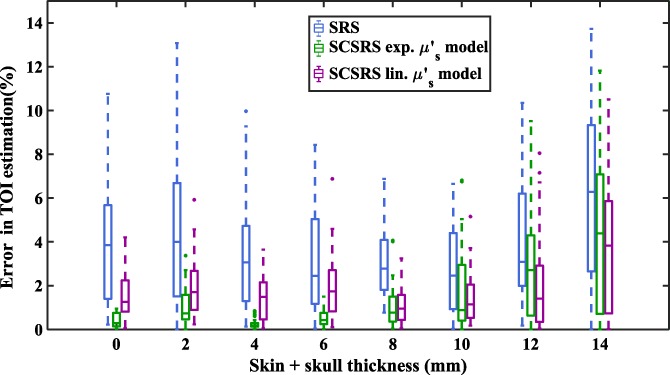
Boxplot of absolute error in the estimation of tissue oxygenation index (TOI) for different skin + skull thicknesses, showing its distribution across different ground‐truth TOI and scattering parameters

To simulate the effect of different tissue thicknesses, a rectangular‐slab finite element method model (200 × 100 × 100 mm^3^) of mean element size of 0.7 mm^3^, is considered with three layers corresponding to the three layers of human head: skin, skull and brain. The total thickness of skin and skull is varied from 0 to 14 mm in steps of 2 mm to simulate possible tissue thicknesses from an infant head 29 to an adult head while the ratio of skin to skull thickness is maintained 5:8 in accordance with the average thickness of real head models considered in Section 4. Using the medium properties as defined in Table 1, the transmitted light intensity data is simulated on NIRFAST. The modeled measurement system is similar to the one considered in previous section with one source and two detectors (37 and 43 mm from the source) at 735, 810 and 850 nm wavelengths. To account for the inter‐subject variability the data is simulated for 30 different cases with scattering properties randomly varied around the baseline values given in Table 1, by a SD of 30% and TOI values of brain region is changed from 50% to 80%. The recovery of tissue oxygenation is shown in Figure 9 for different possible superficial layer thicknesses along with the error plots shown in Figure 10. The conventional SRS method is implemented using a linear scatter model with *b* = 0.63 μm^−1^ corresponding to an adult head.

For a homogeneous medium (ie, skin + skull thickness = 0 mm), the SCSRS method with both scattering models exhibit a mean error of less than 2% out‐performing the SRS method which results in errors up to 6%. The lower errors for the SCSRS with exponential scattering model as compared to the linear scattering model is to be attributed to the fact that the data was simulated using exponential scattering model and this advantage seems to be prevalent only up to a superficial layer thickness of 8 mm where the inhomogeneity starts to dominate. The decrease in overall mean errors in TOI estimation for superficial thickness 4 and 6 mm is due to the higher TOI values of superficial layer. It can be seen clearly from Figure 9 that as the superficial layer thickness increases from 0 to 14 mm, the estimated cerebral mean TOI values demonstrate an underestimation (negative errors) and tend to move towards the TOI of the superficial layers (80%, shown in Table 1), which is an overestimation (positive errors) for cerebral TOI (50% to 80%, shown in Table 1). This shift from negative (underestimation) to positive (overestimation) errors causes the absolute errors to reach lower values, which is seen to be happening at superficial layer thicknesses of 4 and 6 mm. At higher thicknesses of superficial layers the mean TOI recovery tends to be inaccurate and the recovery errors for SCSRS increase up to 7.7% with an exponential scattering model and 5.8% with a linear scattering model, which are still lower than a recovery error of 9.3% using conventional SRS. All the quantitative errors quoted above are the 75th percentile errors from Figure 10. These results demonstrate that the proposed method of SCSRS, with both exponential scatter model and linear scatter model, performs better than the conventional SRS.

## 6 ARM‐CUFF EXPERIMENT

**Figure 11:**
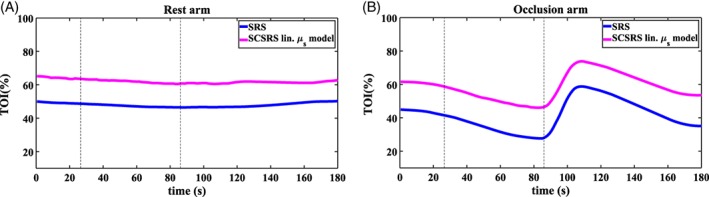
Tissue oxygenation index measurement on human forearm: Rest arm recovered b‐value from SCSRS is 0.79 ± 0.007 μm^−1^, and Occlusion arm recovered b‐value from Spectrally Constrained Spatially Resolved Spectroscopy is 0.82 ± 0.003 μm^−1^

The proposed SCSRS method is tested on experimental data from forearm occlusion experiment (institutional ethical approval: ERN16‐1490) using a commercial CW‐system, the NIRO‐200NX. The probes were attached to both forearms of a human subject and a blood pressure cuff was applied on the upper arm with the cuff inflated to 140 mm Hg for 60 seconds to observe arterial occlusion. The conventional SRS is also implemented (linear $\mu_{s}^{\prime }$ model: *b* = 0.47 μm^−1^ corresponding an adult forearm 30) to compare the results with SCSRS, Figure 11. Both methods show a similar trend in the changes of recovered TOI, but the absolute values differ by more than 10%.

The recovered values of “b” with SCSRS were 0.79 ± 0.007 and 0.82 ± 0.003 μm^−1^ for each arm respectively. Although these are similar for each arm, they are different as compared to the average literature value of 0.47 μm^−1^ (as used for SRS algorithm) 30. Since the variation in the *b* parameter across different subjects and tissues can be large 18, 30, the proposed SCSRS method overcomes this by fitting for a *b* parameter and simultaneously recovering the TOI using the optimal wavelengths. Finally, although the proposed algorithms provide a TOI which is higher than the SRS method, this is likely to be more accurate based on results in Figure 9, where the SRS always under‐estimates the recovered TOI.

## 7 DISCUSSION AND CONCLUSION

A CW NIRS parameter recovery algorithm is presented to estimate the TOI without any prior assumptions on the tissue scatter properties. The two major challenges for a CW system are the signal contamination from superficial layers as well as the separation of absorption and scattering information. It is shown that the SCSRS method which is based on gradient of light of attenuation measurement is less sensitive to superficial region and more sensitive to deeper tissues. Although the superficial layer contamination is shown to be minimized using the gradient measurement, the error due to the heterogeneity of a layered medium still exists and is shown to cause an underestimation of the recovered TOI values of the brain.

The parameter recovery involves inversion of a Jacobian matrix, for which an optimal set of wavelengths (734 ± 25, 805 ± 6 and 848 ± 2 nm) have been found to maximize the information present while minimizing the crosstalk between parameters. This is similar to previous wavelength optimization work done by Eames et al 28 where they recovered optimal wavelengths which were also based on normalization of the absorption by the scattering properties (ie, an assumed pathlength). It should however be noted that central wavelengths of interest are always a function of the absorption parameters being recovered and although the methodology can be generalized this should be repeated for each specific application under investigation.

The inter‐subject variability in the scattering properties is known to be significant, which has been identified through findings in other works 18, 30. Therefore, any assumption of the scattering properties for the estimation of absorbing properties such as the tissue oxygenation would result in an uncertain estimation, which is the case with SRS. The feasibility of the proposed SCSRS approach is shown through simulations, to measure the deep layer tissue oxygenation with higher accuracy (average recovery errors of <5%) by simultaneously fitting the scattering parameters for adult head geometries. The repeatability (low variation) of recovered scattering parameters for different cerebral TOIs (50% to 80%) on the same subject, as shown in Figure 8, demonstrates that the cross‐talk between the recovered parameters is low which can be attributed to the wavelength optimization. Variation of 50% to 80% TOI in brain tissue is considered as safe range beyond which it is regarded as cerebral hypoxia, in which case clinical intervention is opted 31, 32. This work is therefore only considered with the range of values with these practical extreme values. The accuracy of the TOI recovery, as demonstrated, decreases with lower brain TOI values due to the increased heterogeneity, therefore it is expected that for lower TOIs it follows a similar trend.

The proposed SCSRS method has been applied to an arm‐cuff experiment with the obtained TOI compared to those of conventional SRS (where the tissue scattering is assumed a‐priori). It is shown that although both methods provide a similar change in TOI variation due to the applied pressure, the absolute value of the obtained TOI for SCSRS is more realistic (eg, approximately 65% as compared to approximately 50% for resting state), while also accounting for the unknown tissue scattering. Although validation of this is challenging, as the absolute TOI of tissue is unknown, it is the subject of future studies and needs further investigation.

Finally, although the heterogeneous nature of the head limits the accuracy of this method, the proposed SCSRS is shown to outperform the conventional SRS method. The advantage of this method is that it is compatible with the commercially available devices such as NIRO‐200NX without any additional instrumentational changes.

## AUTHOR BIOGRAPHIES

Please see Supporting Information online.

## Supporting information

Supporting Information
