# Supplementary material for: Functional near infrared spectroscopy using spatially resolved data to account for tissue scattering: A numerical study and arm‐cuff experiment
Source: J Biophotonics. 2019 Jun 23;12(10):e201900064. doi: 10.1002/jbio.201900064 (PMC7065609; doi:10.1002/jbio.201900064)
Supplement: Supplementary file 1 — Supporting Information [file JBIO-12-e201900064-s001.docx]

Author biographies:

| 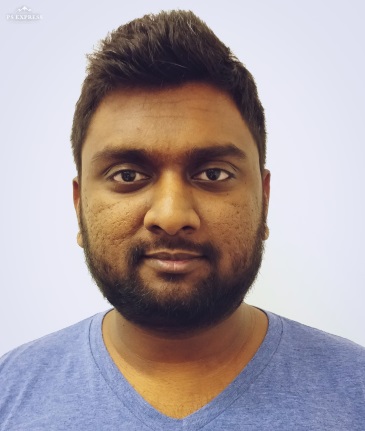 | **Joshua Deepak Veesa** received his M.Tech degree in optical engineering from Indian Institute of Space Science and Technology, Trivandrum, India. Currently, he is a Ph.D. student at the School of Computer Science, University of Birmingham, United Kingdom. His research focuses on developing computational models in near-infrared spectroscopy to monitor brain health. |
| --- | --- |
| 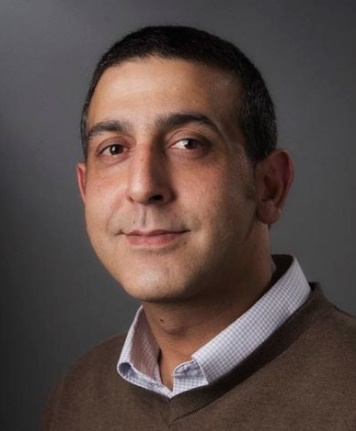 | **Hamid Dehghani** is the Professor of Medical Imaging in the School of Computer Science at the University of Birmingham, Birmingham, United Kingdom and an OSA Fellow. He has published over 100 peer reviewed journal papers in the area of image reconstruction and numerical modelling and has a long and established track record in the development of biophotonics based techniques with specific applications in in-vivo optical imaging. |
